# CDC Recommendations for Hepatitis C Screening Among Adults — United States, 2020

**DOI:** 10.15585/mmwr.rr6902a1

**Published:** 2020-04-03

**Authors:** Sarah Schillie, Carolyn Wester, Melissa Osborne, Laura Wesolowski, A. Blythe Ryerson

**Affiliations:** 1 Division of Viral Hepatitis, National Center for HIV/AIDS, Viral Hepatitis, STD, and TB Prevention, CDC

## Abstract

Hepatitis C virus (HCV) infection is a major source of morbidity and mortality in the United States. HCV is transmitted primarily through parenteral exposures to infectious blood or body fluids that contain blood, most commonly through injection drug use. No vaccine against hepatitis C exists and no effective pre- or postexposure prophylaxis is available. More than half of persons who become infected with HCV will develop chronic infection. Direct-acting antiviral treatment can result in a virologic cure in most persons with 8–12 weeks of all-oral medication regimens. This report augments (i.e., updates and summarizes) previously published recommendations from CDC regarding testing for HCV infection in the United States (*Smith BD, Morgan RL, Beckett GA, et al. Recommendations for the identification of chronic hepatitis C virus infection among persons born during 1945–1965. MMWR Recomm Rec 2012;61[No. RR-4]*). CDC is augmenting previous guidance with two new recommendations: 1) hepatitis C screening at least once in a lifetime for all adults aged ≥18 years, except in settings where the prevalence of HCV infection is <0.1% and 2) hepatitis C screening for all pregnant women during each pregnancy, except in settings where the prevalence of HCV infection is <0.1%. The recommendation for HCV testing that remains unchanged is regardless of age or setting prevalence, all persons with risk factors should be tested for hepatitis C, with periodic testing while risk factors persist. Any person who requests hepatitis C testing should receive it, regardless of disclosure of risk, because many persons might be reluctant to disclose stigmatizing risks.

## Introduction

Hepatitis C is the most commonly reported bloodborne infection in the United States (*1*), and surveys conducted during 2013*–*2016 indicated an estimated 2.4 million persons (1.0%) in the nation were living with hepatitis C (*2*). Percutaneous exposure is the most efficient mode of hepatitis C virus (HCV) transmission, and injection drug use (IDU) is the primary risk factor for infection (*1*). National surveillance data revealed an increase in reported cases of acute HCV infection every year from 2009 through 2017 (*1*). The highest rates of acute infection are among persons aged 20*–*39 years (*1*). As new HCV infections have increased among reproductive aged adults, rates of HCV infection nearly doubled during 2009*–*2014 among women with live births (*3*). In 2015, 0.38% of live births were delivered by mothers with hepatitis C (*4*).

This report augments (i.e., updates and summarizes) previous CDC recommendations for testing of hepatitis C among adults in the United States published in 1998 and 2012 (*5*,*6*). The recommendations in this report do not replace or modify previous recommendations for hepatitis C testing that are based on known risk factors or clinical indications. Previously published recommendations for hepatitis C testing of persons with risk factors and alcohol use screening and intervention for persons identified as infected with HCV remain in effect (*5*,*6*). This report is intended to serve as a resource for health care professionals, public health officials, and organizations involved in the development, implementation, delivery, and evaluation of clinical and preventive services.

### Epidemiology

**FIGURE 1:**
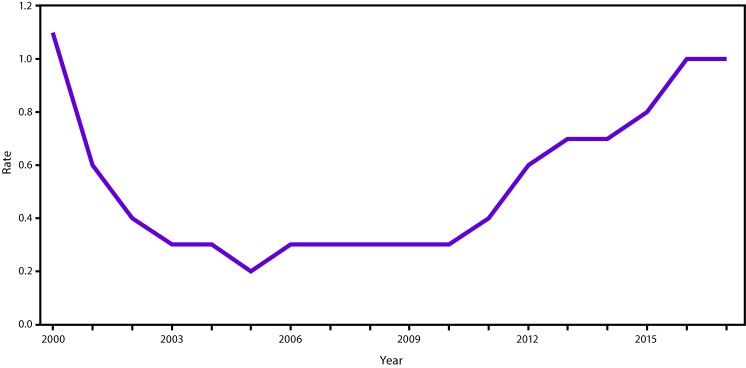
Incidence rates* of reported acute hepatitis C cases ── United States, 2000–2017 * Per 100,000 population.

Source: CDC, National Notifiable Diseases Surveillance System.

In 2017, a total of 3,216 cases (1.0 per 100,000 population) of acute HCV infection were reported to CDC (*1*). The reported number of cases in any given year likely represents less than 10% of the actual number of cases because of underascertainment and underreporting (*7*). An estimated 44,700 new cases of HCV infection occurred in 2017. The rate of reported acute HCV infections increased from 0.7 cases per 100,000 population in 2013 to 1.0 in 2017 (Figure 1) (*1*). In 2017, acute HCV incidence was greatest for persons aged 20*–*29 years (2.8) and 30*–*39 years (2.3) (*1*). Persons aged ≤19 years had the lowest incidence (0.1) (*1*). Incidence was slightly greater for males than females (1.2 cases and 0.9, respectively) (*1*). During 2006*–*2012, the combined incidence of acute HCV infection in four states (Kentucky, Tennessee, Virginia, and West Virginia) increased 364% among persons aged ≤30 years. Among cases in these states with identified risk information, IDU was most commonly reported (73%). Those infected were primarily non-Hispanic white persons from nonurban areas (*8*).

**FIGURE 2:**
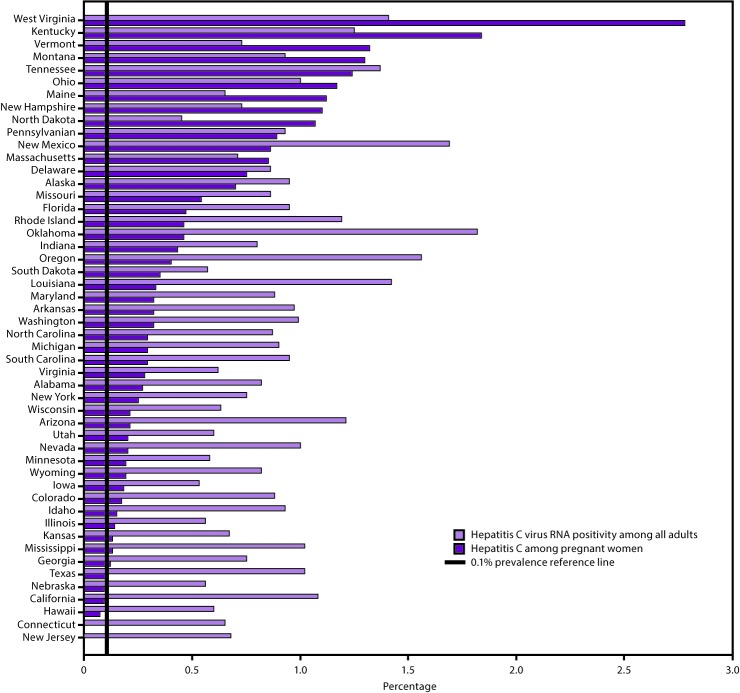
Estimated prevalence of hepatitis C virus RNA positivity among all adults* and hepatitis C among pregnant women,^†^ by state**^§^** **Abbreviations:** HCV = hepatitis C virus; RNA = ribonucleic acid; NHANES = National Health and Nutrition Examination Survey. * State estimates of HCV RNA positivity among all adults are based on a statistical model that allocated nationally representative hepatitis C prevalence from the 2013–2016 NHANES and additional previously published data for populations not sampled in NHANES to states according to the spatial demographics and distributions of 1999–2016 hepatitis C mortality and narcotic overdose deaths in the National Vital Statistics System. ^†^ Data are from an analysis of 2015, National Center for Health Statistics birth certificate data (live births) (Schillie SF, Canary L, Koneru A, et al. Hepatitis C virus in women of childbearing age, pregnant women, and children. Am J Prev Med 2018;55:633–41). ^§^ Connecticut did not report maternal HCV infection on 2015 birth certificates and New Jersey reported infections from only a limited number of facilities; therefore, women residing in these two states were not included in the analysis.

On the basis of 2013*–*2016 National Health and Nutrition Examination Survey (NHANES) data and considering populations not sampled in NHANES, an estimated 1.0% of all adults in the United States, or 2,386,100 persons, were living with HCV infection (HCV RNA positive) (*2*). Nine states comprise 51.9% of all persons living with HCV infection: California, Florida, New York, North Carolina, Michigan, Ohio, Pennsylvania, Tennessee, and Texas (Figure 2) (*9*).

### Virus Description and Transmission

HCV is a small, single-stranded, enveloped RNA virus in the flavivirus family with a high degree of genetic heterogeneity. Seven distinct HCV genotypes have been identified. Genotype 1 is the most prevalent genotype in the United States and worldwide, accounting for approximately 75% and 46% of cases, respectively (*10*,*11*). Geographic differences in global genotype distribution are important because some treatment options are genotype specific (*11*,*12*). High rates of mutation in the HCV RNA genome are believed to play a role in the pathogen’s ability to evade the immune system (*11*). Prior infection with HCV does not protect against subsequent infection with the same or different genotypes.

HCV is primarily transmitted through direct percutaneous exposure to blood. Mucous membrane exposures to blood also can result in transmission, although this route is less efficient. HCV can be detected in saliva, semen, breast milk, and other body fluids, although these body fluids are not believed to be efficient vehicles of transmission (*11*,*13*).

### Persons at Risk for HCV Infection

IDU is the most common means of HCV transmission in the United States. Invasive medical procedures (e.g., injections and hemodialysis) pose risks for HCV infection when standard infection-control practices are not followed (*14*,*15*). Health care*–*related hepatitis C outbreaks also stem from drug diversion (e.g., tampering with fentanyl syringes) (*16*,*17*). Although HCV infection is primarily associated with IDU, high-risk behaviors (e.g., anal sex without using a condom), primarily among persons with HIV, are also important risk factors for transmission (*18*). Other possible exposures include sharing personal items contaminated with blood (e.g., razors or toothbrushes), unregulated tattooing, needlestick injuries among health care personnel, and birth to a mother with hepatitis C. Receipt of donated blood, blood products, and organs was once a common means of transmission but is now rare in the United States (*19*).

Before implementing universal blood product testing in 1992, children acquired hepatitis C predominantly through blood transfusion. Because of the increasing incidence of HCV infection among women of childbearing age, perinatal transmission (intrauterine or intrapartum) has become an increasingly important mode of HCV transmission (*20*,*21*). Among pregnant women from 2011 to 2016, hepatitis C virus testing increased by 135% (from 5.7% to 13.4%), and positivity increased by 39% (from 2.6% to 3.6%) (*4*). The risk for perinatal transmission is informed by a systematic review and meta-analysis of studies conducted in multiple countries and is 5.8% for infants born to mothers infected with HCV but not with HIV and doubles for infants born to mothers co-infected with HCV and HIV. Perinatal HCV transmission is almost always confined to infants born to mothers with detectable HCV RNA (*22*). Only approximately 20% of infants with perinatally acquired hepatitis C clear the infection, 50% have chronic asymptomatic infection, and 30% have chronic active infection (*23*). HCV-related liver disease rarely causes complications during childhood. Because fibrosis increases with disease duration, perinatally infected persons might develop severe disease as young adults (*20*,*21*).

### Clinical Features and Natural History

Persons with acute HCV infection are typically either asymptomatic or have a mild clinical illness like that of other types of viral hepatitis (*24*). Jaundice might occur in 20%–30% of persons, and nonspecific symptoms (e.g., anorexia, malaise, or abdominal pain) might be present in 10%–20% of persons. Fulminant hepatic failure following acute hepatitis C is rare. The average time from exposure to symptom onset is 2–12 weeks (range: 2–26 weeks) (*25*,*26*). HCV antibodies (anti-HCV) can be detected 4–10 weeks after infection and are present in approximately 97% of persons by 6 months after exposure. HCV RNA can be detected as early as 1–2 weeks after exposure. The presence of HCV RNA indicates current infection (*27*–*29*).

Historically, approximately 15%–25% of persons were believed to resolve their acute infection without sequelae (*30*); however, more recent data suggest that spontaneous clearance might be as high as 46%, varying by age at the time of infection (*31*). Spontaneous clearance is lower among persons co-infected with HIV (*11*). Predictors of spontaneous clearance include jaundice; elevated alanine aminotransferase (ALT) level; hepatitis B virus surface antigen (HBsAg) positivity; female sex; younger age; HCV genotype 1; and host genetic polymorphisms, most notably those near the IL28B gene (*27*–*29*). Chronic HCV infection develops when viral replication evades the host immune response. The course of chronic liver disease is usually insidious, progressing slowly without symptoms or physical signs in most persons during the first 20 years or more following infection. Approximately 5%–25% of persons with chronic hepatitis C will develop cirrhosis over 10–20 years (*30*). Those with cirrhosis experience a 1%–4% annual risk for hepatocellular carcinoma (*30*). Persons who are male, aged >50 years, use alcohol, have nonalcoholic fatty liver disease, have hepatitis B virus (HBV) or HIV coinfection, and who are undergoing immunosuppressive therapy have increased rates of progression to cirrhosis. Extrahepatic manifestations of chronic HCV infection might occur and include membranoproliferative glomerulonephritis, essential mixed cryoglobulinemia, porphyria cutanea tarda (*27*–*29*), and lymphoma (*32*).

### Diagnosis and Hepatitis C Elimination

In one report, the National Academies of Sciences, Engineering, and Medicine explored the feasibility of hepatitis C elimination and concluded that hepatitis C could be eliminated as a public health problem in the United States, but that substantial obstacles exist (*33*). In another report, specific actions were recommended to achieve elimination considering information, interventions, service delivery, financing, and research (*34*). These reports were the culmination of decades of progress in the development of HCV infection diagnostic and therapeutic tools.

In 1990, serologic tests to detect immunoglobulin G anti-HCV by enzyme immunoassay were licensed and became commercially available in the United States, and U.S. blood banks voluntarily began testing donations for anti-HCV (*35*). In 1991, U.S. Public Health Service issued interagency guidelines addressing hepatitis C screening of blood, organs, and tissues (*35*). These guidelines recommended hepatitis C testing for all donations of whole blood and components for transfusion, as well as testing serum/plasma from donors of organs, tissues, or semen intended for human use (*35*).

In 1998, CDC expanded the interagency guidelines to provide recommendations for preventing transmission of HCV; identifying, counseling, and testing persons at risk for hepatitis C; and providing appropriate medical evaluation and management of persons with hepatitis C (*6*). The guidelines recommended testing on the basis of risk factors for HCV infection for persons who ever injected drugs and shared needles, syringes, or other drug preparation equipment, including those who injected once or a few times many years ago and do not consider themselves as drug users; with selected medical conditions, including those who received clotting factor concentrates produced before 1987; who were ever on chronic hemodialysis (maintenance hemodialysis); with persistently abnormal ALT levels; who were prior recipients of transfusions or organ transplants, including those who were notified that they received blood from a donor who later tested positive for HCV infection; who received a transfusion of blood or blood components before July 1992, or who received an organ transplant before July 1992; and with a recognized exposure, including health care, emergency medical, and public safety workers after a needlestick injury, sharps injury, or mucosal exposure to blood infected with hepatitis C or children born to mothers infected with hepatitis C (*6*). In 1999, the U.S. Public Health Service and Infectious Diseases Society of America (IDSA) guidelines recommended hepatitis C testing for persons with HIV (*36*).

Because of the limited effectiveness of risk-based hepatitis C testing, CDC considered strategies to increase the proportion of infected persons who are aware of their status and are linked to care (*5*). In 2012, CDC augmented its guidance to recommend one-time hepatitis C screening for persons born during 1945–1965 (birth cohort) without ascertainment of risk (*5*). With an anti-HCV positivity prevalence of 3.25%, persons born in the 1945–1965 birth year cohort accounted for approximately three fourths of chronic HCV infections among U.S. adults during 1999–2008 (*37*). Approximately 45% of persons infected with HCV do not recall or report having specific risk factors (*38*). Included in the 2012 guidelines were recommendations for alcohol use screening and intervention for persons identified with HCV infection (*5*). This report expands hepatitis C screening to at least once in a lifetime for all adults aged >18 years, except in settings where the prevalence of HCV infection is <0.1%.

The 2012 CDC guidelines recommended that pregnant women be tested for hepatitis C only if they have known risk factors (*5*). However, in 2018, universal hepatitis C screening during pregnancy was recommended by the American Association for the Study of Liver Diseases and IDSA (*39*). This report expands hepatitis C screening for all pregnant women during each pregnancy, except in settings where the prevalence of HCV infection is <0.1%.

Existing strategies for hepatitis C testing have had limited success. The 2013–2016 surveys indicate only approximately 56% of persons with HCV infection reported having ever been told they had hepatitis C (*38*). Therefore, strengthened guidance for universal hepatitis C testing is warranted. Models to address barriers related to access to direct-acting antiviral (DAA) treatment are needed to ensure health care equity and the success of expanded hepatitis C screening. The recommendation for HCV testing that remains unchanged is regardless of age or setting prevalence, all persons with risk factors should be tested for hepatitis C, with periodic testing while risk factors persist. Any person who requests hepatitis C testing should receive it regardless of disclosure of risk because many persons might be reluctant to disclose stigmatizing risks.

### Clinical Management and Treatment

The treatment for HCV infection has evolved substantially since the introduction of DAA agents in 2011. DAA therapy is better tolerated, of shorter duration, and more effective than interferon-based regimens used in the past (*39*,*40*). The antivirals for hepatitis C treatment include next-generation DAAs, categorized as either protease inhibitors, nucleoside analog polymerase inhibitors, or nonstructural (NS5A) protein inhibitors. Many agents are pangenotypic, meaning they have antiviral activity against all genotypes (*20*,*21*,*40*). A sustained virologic response (SVR) is indicative of cure and is defined as the absence of detectable HCV RNA 12 weeks after completion of treatment. Approximately 90% of HCV-infected persons can be cured of HCV infection with 8–12 weeks of therapy, regardless of HCV genotype, prior treatment experience, fibrosis level, or presence of cirrhosis (*39*–*41*).

Despite their favorable safety profile, DAAs are not yet approved for use in pregnancy. Safety data during pregnancy are preliminary and larger studies are required. A small study of seven pregnant women treated with ledipasvir/sofosbuvir identified no safety concerns (*42*). Until DAAs become available for use during pregnancy, testing women during pregnancy for HCV infection still has benefits to both the mother and the infant. Many women only have access to health care during pregnancy and the immediate postpartum period. In 2017, 12.4% of women aged 19–44 years were not covered by public or private health insurance (*43*). Pregnancy is an opportune time for women to receive a hepatitis C test while simultaneously receiving other prenatal pathogen testing such as for HIV or hepatitis B. The postpartum period might represent a unique time to transition women who have had HCV infection diagnosed during pregnancy to treatment with DAAs. Treatment during the interconception (interpregnancy) period reduces the transmission risk for subsequent pregnancies. Identification of HCV infection during pregnancy also can inform pregnancy and delivery management issues that might reduce the likelihood of HCV transmission to the infant. The Society for Maternal-Fetal Medicine recommends a preference for amniocentesis over chorionic villus sampling when needed, and for avoiding internal fetal monitoring, prolonged rupture of the membranes, and episiotomy among HCV-infected women, unless it is unavoidable (*44*).

Testing during pregnancy allows for simultaneous identification of infected mothers and infants who should receive testing at a pediatric visit. Testing of infants consists of HCV RNA testing at or after age 2 months or anti-HCV testing at or after age 18 months (*39*). Although DAA treatment is not approved for children aged <3 years, infected children aged <3 years should be monitored. In 2017, ledipasvir/sofosbuvir became the first DAA approved for use in persons aged 12–17 years (*20*,*21*). In 2019 glecaprevir/pibrentasvir became approved for use in persons aged ≥12 years (*45*), and ledipasvir/sofosbuvir became approved for use in persons aged ≥3 years (*46*).

No vaccine against hepatitis C exists and no effective pre- or postexposure prophylaxis (e.g., immune globulin) is available. Prenatal treatment options and/or infant antiviral postexposure prophylaxis might become available to prevent perinatal transmission. HCV infection is not an indication for Cesarean delivery and is not a contraindication to breastfeeding if nipples are not bleeding or cracked (*44*).

## Methods

To inform these recommendations, comprehensive systematic reviews of the literature were conducted, analyzed, and assessed in two stages. These reviews examined the availability of evidence regarding HCV infection prevalence and the health benefits and harms associated with one-time hepatitis C screening for persons unaware of their status.

CDC determined that the new recommendations constituted scientific information that will have a clear and substantial impact on important public policies and private sector decisions. Therefore, the Information Quality Act required peer review by specialists in the field who were not involved in the development of these recommendations. CDC solicited nominations for reviewers from the American Association for the Study of Liver Diseases (AASLD), IDSA, and the American College of Obstetricians and Gynecologists (ACOG). Six clinicians with expertise in hepatology, gastroenterology, internal medicine, infectious diseases and/or obstetrics and gynecology provided structured peer reviews. In addition, feedback from the public was solicited through a Federal Register notice released on October 28, 2019, announcing the availability of the draft recommendations for public comment through December 27, 2019. CDC received 69 public comments on the draft document from academia, professional organizations, industry, and the public. Many of the comments from both peer reviewers and the public were in support of the recommendations. For those comments that proposed changes, the majority related to screening for hepatitis C in every pregnancy or removing the prevalence threshold for universal screening. Feedback attained during both the peer review process and the public comment period was reviewed by CDC. Ultimately, no changes to the recommendations were made; however, additional references and justification for the recommendation to screen during every pregnancy and maintaining the prevalence threshold were added to the document.

To facilitate the systematic review of the evidence, two research questions were formulated to guide the development of the recommendations:

- Does universal screening for hepatitis C virus infection among adults aged ≥18 years, compared with risk-based screening, reduce morbidity and mortality?
- Does universal screening for hepatitis C virus infection among pregnant women, compared with risk-based screening, reduce morbidity and mortality among mothers and their children?

An analytic framework describing the chain of indirect evidence was developed:

- How would universal screening for hepatitis C affect the number (and composition) of persons who screen positive for HCV infection?
- How many additional persons would be linked to care?
- Do desirable treatment effects outweigh undesirable effects?

Key questions (KQ) were formulated for each link of the chain (Supplementary Table 1, https://stacks.cdc.gov/view/cdc/85840):

- K.Q.1.a. What is the prevalence of HCV infection in the United States in the general population and by risk groups?
- K.Q.2.a. What is the diagnostic accuracy of HCV antibody testing?
- K.Q.2.b. What are the harms of hepatitis C screening?
- K.Q.2.c. What proportion of persons who screen positive for HCV infection are linked to care?
- K.Q.3.a. What is the effect of DAA treatment on HCV viral load?
- K.Q.3.b. What is the effect of DAA treatment on morbidity (including cirrhosis and hepatocellular carcinoma)?
- K.Q.3.c. What is the effect of DAA treatment on mortality (HCV-specific and all-cause)?
- K.Q.3.d. What are the adverse effects of DAA treatment?

Because the diagnostic accuracy of anti-HCV testing and treatment effects have been described previously, K.Q.2.a. and K.Q.3.a.–d. key questions were not included in this review.

### Literature Review

Systematic reviews were conducted to examine benefits and harms of hepatitis C screening. The systematic review process for these recommendations was separated into two stages: 1) a review of evidence to inform the hepatitis C screening strategy among all adults and 2) a review of the evidence to inform the hepatitis C screening strategy among pregnant women.

Systematic reviews were conducted for literature published worldwide in Medline (OVID), Embase (OVID), CINAHL (EBSCO), Scopus, and Cochrane Library. For the all-adult review, the beginning search date was 2010 to capture studies reflecting the changing epidemiology of HCV infection and the availability of DAAs, and the end date was the run date of August 6, 2018 (Supplementary Table 2, https://stacks.cdc.gov/view/cdc/85840). For the pregnancy review, the beginning search date was 1998 to capture studies published since previous recommendations were issued in 1998, and the end date was the run date of July 2, 2018 (Supplementary Table 3, https://stacks.cdc.gov/view/cdc/85840). Duplicates were identified using the Endnote (Clarivate Analytics, Philadelphia, Pennsylvania, United States) automated “find duplicates” function with preference set to match on title, author, and year. Duplicates were removed from the Endnote library.

Following the initial collection of results from the search, titles/abstracts were independently reviewed by two persons. For papers in which the title indicated the study was irrelevant to the research question, abstracts were not reviewed.

Titles/abstracts for the all-adult review were independently reviewed by two reviewers, one of whom was always a senior abstractor (and author LW or SS). Conflicts were resolved by SS. If a conflict arose from a study whose title/abstract was reviewed only by both LW and SS, that study was retrieved for the full text review. All full texts were screened by both MO and LW. SS made the final decision regarding conflicts. Information from the full texts was extracted for the evidence review. A systematic review software program (Covidence; Melbourne, Victoria, Australia) was used to facilitate the all-adult review process.

Titles/abstracts for the pregnancy review were independently reviewed by two senior abstractors (LW or SS). Studies that either abstractor deemed as potentially relevant were retrieved for full text review. All full texts were screened by both senior abstractors. Information from the full texts was extracted for the evidence review.

Studies were excluded if they were conducted in a correctional facility because separate CDC guidance for hepatitis C screening in correctional facilities is under development. Other reasons for exclusion were: if prevalence data from 2010 forward could not be abstracted (all-adult review only); if the study reported estimated, projected, or self-reported data; if data were only available from a conference abstract, or if the study population was non-U.S. based, unless the study examined outcomes related to harms of screening. Studies related to harms of screening were included broadly to help ensure all potential harms were captured in the review. When multiple studies reported data for the same patients (e.g., when results of an initial pilot study were reported or when multiple studies reported outcomes of the CDC-funded Hepatitis Testing and Linkage to Care Project) (*47*), only the study with the most complete data was included. Linkage-to-care data were abstracted from 2010 forward from studies formally assessing linkage-to-care and reporting arrangement of or attendance at a follow-up appointment with a provider with special training for hepatitis C management. HCV RNA testing alone was not deemed linkage-to-care for purposes of this review, and studies did not have to report achievement of SVR to be included in the linkage-to-care review. Study design and setting were abstracted for all applicable studies. After the formal literature review was conducted, relevant studies identified through reference lists and those that were newly published were added for review.

To capture recently published studies, a supplementary literature search was conducted on November 15, 2019 for all adults (Supplementary Table 4, https://stacks.cdc.gov/view/cdc/85840) and on October 29, 2019 for pregnant women (Supplementary Table 5, https://stacks.cdc.gov/view/cdc/85840). The search strategy was the same as for the original searches. Titles/abstracts were independently reviewed by BR and SS. In the case of a conflict, the study was kept for full text review. Full texts were independently reviewed by two reviewers, one of whom was either MO, BR, or SS for the all-adult review and BR or SS for the pregnant women review. Information from the full texts was abstracted and added to the original review.

### Summary of the Literature

For the all-adult review, the initial literature search yielded 4,867 studies. Twenty-nine duplicates were identified. Of 4,838 unique studies, 4,170 (86.2%) were deemed irrelevant by title/abstract screening, resulting in 668 (13.8%) full texts for review. Among these, 368 studies had data available to extract.

For the pregnancy review, the initial literature search yielded 1,500 studies. Two duplicates were identified. Of 1,498 unique studies, 1,412 (94.3%) were deemed irrelevant by title/abstract screening, resulting in 86 (5.7%) full texts for review.

The supplementary review yielded an additional 1,038 and 195 studies among all adults and pregnant women, respectively. Of these, 912 (87.9%) and 168 (86.2%), respectively, were deemed irrelevant by title/abstract screening, resulting in 126 (12.1%) and 27 (13.9%), respectively, full texts for review. One study was added to the pregnant women review outside of the formal literature search (i.e., the study was not among the retrieved studies but was known by the authors) (*3*).

Considering all 104 applicable studies, the median anti-HCV positivity prevalence (indicative of past or current infection) among all adults was 6.6% (range: 0.0%–76.1%) (Table). Median anti-HCV positivity prevalence was 1.7% (range: 0.02%–7.9%) for the general population (nine studies) (Supplementary Table 6, https://stacks.cdc.gov/view/cdc/85840), 7.5% (range: 0.5%–25.8%) for ED patients (19 studies) (Supplementary Table 7, https://stacks.cdc.gov/view/cdc/85840), 3.3% (range: 0.0%–43.5%) for birth cohort members (31 studies) (Supplementary Table 8, https://stacks.cdc.gov/view/cdc/85840), 9.3% (range: 1.6%–76.1%) for others/multiple risk factors (23 studies) (Supplementary Table 9, https://stacks.cdc.gov/view/cdc/85840), 54.2% (range: 12.7%–67.1%) for persons who use drugs (11 studies) (Supplementary Table 10, https://stacks.cdc.gov/view/cdc/85840), 5.2% (range: 1.2%–32.9%) for persons with HIV or sexual risk (eight studies) (Supplementary Table 11, https://stacks.cdc.gov/view/cdc/85840), and 4.7% (range: 3.4%–7.5%) for immigrants (three studies) (Supplementary Table 12, https://stacks.cdc.gov/view/cdc/85840). Considering 26 applicable studies among pregnant women, median anti-HCV positivity prevalence was 1.2% (range: 0.1%–70.8%) (Supplementary Table 13, https://stacks.cdc.gov/view/cdc/85840).

**TABLE:** Hepatitis C prevalence by adult populations, summary of literature review

Population	No. studies informing anti-HCV positivity prevalence	Median anti-HCV prevalence (range)	No. studies informing HCV RNA positivity prevalence	Median HCV RNA positivity (range)
**All studies**	**104**	**6.6% (0.0%–76.1%)**	**61**	**68.7% (20.0%–100.0%)**
General population	9	1.7% (0.02%–7.9%)	6	55.2% (36.8%–83.0%)
Emergency department patients	19	7.5% (0.5%–25.8%)	12	69.0% (42.5%–90.5%)
Birth cohort	31	3.3% (0.0%–43.5%)	21	62.7% (20.0%–95.3%)
Others/multiple	23	9.3% (1.6%–76.1%)	14	74.1% (47.0%–100.0%)
Persons who use drugs	11	54.2% (12.7%–67.1%)	3	73.8% (69.9%–100.0%)
Persons with HIV or sexual risks	8	5.2% (1.2%–32.9%)	4	63.4% (41.4%–83.8%)
Immigrants	3	4.7% (3.4%–7.5%)	1	81.8%

**Abbreviations:** anti-HCV = hepatitis C virus antibody; HCV = hepatitis C virus; NHANES = National Health and Nutrition Examination Survey; RNA = ribonucleic acid; SVR = sustained virologic response;

Considering all 61 applicable studies, the median rate of HCV RNA positivity (indicative of viremia) among those who were anti-HCV positive was 68.7% (range: 20.0%–100%) (Table). Median HCV RNA positivity was 55.2% (range: 36.8%–83.0%) for the general population (six studies) (Supplementary Table 6), 69.0% (range: 42.5%–90.5%) for ED patients (12 studies) (Supplementary Table 7), 62.7% (20.0%–95.3%) for birth cohort members (21 studies) (Supplementary Table 8), 74.1% (range: 47.0%–100%) for others/multiple (14 studies) (Supplementary Table 9), 73.8% (range: 69.9%–100%) for persons who use drugs (three studies) (Supplementary Table 10), 63.4% (range: 41.4%–83.8%) for persons with HIV or sexual risk (four studies) (Supplementary Table 11), and 81.8% for immigrants (one study) (Supplementary Table 12). Median HCV RNA positivity was 66.1% (range: 61.3%–77.2%) for pregnant women (four studies) (Supplementary Table 13).

One primary study (*2*) and one follow-up modeling study (*9*) examined nationally representative anti-HCV and HCV RNA data for adults from the 2013–2016 NHANES as well as data from the literature to estimate prevalence among populations not sampled by NHANES. The national estimate for anti-HCV positivity among adults was 1.7% (95% confidence interval [CI] = 1.4–2.0) (*2*). The HCV RNA prevalence estimate among adults was 1.0% (95% CI = 0.8%–1.1%) (*2*). Forty-two studies informed linkage-to-care among adults. Follow-up appointments or referrals were made for a median of 76.0% of HCV RNA positive patients (range: 25%–100%) (23 studies). A median of 73.9% of patients attended their first follow-up appointment (range: 0.0%–100%) (25 studies). This excludes self-reported data and studies that reported patients who were “linked to care” without explicitly stating the patient attended an appointment. A median of 39.0% of those attending a follow-up appointment received treatment (range: 21.5%–76.1%) (13 studies). Among those who received treatment, a median of 85.2% of patients achieved SVR (range: 66.7%–100%) (14 studies) (Supplementary Tables 6–12, https://stacks.cdc.gov/view/cdc/85840). Because DAAs are not approved for use during pregnancy, linkage-to-care was not assessed for pregnant women.

Harms associated with hepatitis C screening were initially informed by 21 and 12 studies from the all-adult and pregnancy review, respectively, including U.S.-based and non-U.S.-based studies. The supplementary literature search identified five studies from the all-adult review and one study from the pregnancy review informing harms. No study compared harms systematically using comparison groups associated with different screening approaches. Harms informed by the all-adult review included physical harms of screening (two studies) (*48*,*49*); anxiety/stress related to testing or waiting for results (five studies) (*49*–*53*); cost (one study) (*54*); anxiety related to receiving positive results (one study) (*55*); interpersonal outcomes (e.g., problems related to family, friends from learning HCV infection status) (five studies) (*51*,*55*–*58*); attitudes toward persons with hepatitis C, including stigma (11 studies) (*49*,*55*,*57*–*65*); time for screening (two studies) (*49*,*66*); and false-positive results, including among left ventricular assist device patients, possibly precluding heart transplantation (six studies) (*67*–*72*). Harms informed by the pregnancy review included physical harms of screening (one study) (*73*), anxiety (five studies) (*74*–*78*), stigma (one study) (*77*), psychological issues (two studies) (*73*,*79*), fears related to sexual relationships (one study) (*80*), legal ramifications and potential loss of infant custody (one study) (*81*), decreased quality of life (one study) (*82*), social repercussions (one study) (*83*), reluctance to disclose illegal risky behaviors because potential impact on mother or newborn (one study) (*84*), expense (two studies) (*78*,*85*), and false-positive results (one study) (*73*). Other plausible harms associated with hepatitis C screening identified outside of these studies (i.e., by subject matter experts, from the peer review process, or among studies not captured through the formal literature review) include harms associated with undergoing a liver biopsy (e.g., pain, bleeding, intestinal perforation, and death), insurability and employability issues, treatment adverse effects, the need to wait or return for test results, difficulty accessing treatment, and unnecessary Cesarean deliveries and unnecessary avoidance of breastfeeding. CDC concluded that identified or potential harms did not outweigh the benefits of screening.

These literature reviews are subject to at least three limitations. First, heterogeneity of individual study results might not be comparable across studies. For example, regarding anti-HCV positivity, some studies reported the proportion of persons testing positive out of the number of persons tested, while other studies reported the total population as the denominator. Other examples of heterogeneity between studies include varying definitions for follow-up (e.g., variations in provider types [specialist versus primary care provider] for which linkage-to-care was considered and varying definitions of “treated” [e.g., treatment initiated versus completed or not specified]). Second, limitations of the included studies also exist and could carry over into the systematic review findings. For example, recall bias and low response rates might have occurred within individual studies, potentially contributing to similar bias in the overall systematic review results. In addition, studies performed in high-burden areas might not be representative of the general populations and could impact external validity of the systematic review. Finally, publication bias might favor publication of studies reporting high disease prevalence, also potentially impacting external validity.

### Cost-Effectiveness Considerations

Certain recent economic analyses provide information on the cost-effectiveness of hepatitis C screening. One analysis determined universal screening for persons aged ≥18 years, using a health care perspective, and yielded an incremental cost-effectiveness ratio (ICER) of $11,378 per quality-adjusted life year (QALY) gained when compared with 1945–1965 birth cohort screening, using a base case hepatitis C prevalence of 2.6% and 0.29% for birth cohort members and nonbirth cohort members, respectively (*86*). ICER remained below $50,000 per QALY gained, a threshold sometimes considered as a cut-off for determining cost-effectiveness, until the anti-HCV positivity prevalence dropped below 0.07% among nonbirth cohort members. Another analysis calculated an ICER of $28,000/QALY gained under a health care perspective for a strategy of screening all persons aged ≥18 years compared with birth cohort screening, with an additional 280,000 cures and 4,400 fewer cases of hepatocellular carcinoma (*87*). When the national hepatitis C prevalence was halved from the base case of 0.84%, ICER increased to $39,400. ICER remained below $100,000 per QALY gained when varying key parameters across broad ranges (e.g., when there was no improvement in quality of life and costs decreased following early-stage cure, when cost of early-stage disease was $0, when treatment costs varied, and when there was no mortality benefit from SVR). A third analysis reported an ICER of $7,900/QALY gained for one-time general population hepatitis C screening of persons aged 20–69 years compared with risk-based screening using a societal perspective and a base case hepatitis C prevalence of 1.6% (*88*). ICER was $5,400/QALY gained for screening persons born during 1945–1965 compared with risk-based screening with a hepatitis C prevalence of 3.3% for persons in the birth cohort. Birth cohort screening dominated general population screening, although the model also included treatment with ribavirin and pegylated interferon; protease inhibitor therapy was modeled for treatment naïve genotype 1 patients at costs ranging from $61,773–$88,248. Studies using higher treatment costs would be expected to calculate ICERs higher than those with lower treatment costs. Several other studies provide similar cost effectiveness estimates of a universal screening strategy for adults, with ICERs ranging from cost saving to $71,000/QALY gained (*89*–*91*).

Analyses focusing on pregnant women have yielded similar results. One analysis calculated an ICER of $2,826 for universal screening of pregnant women under the health care perspective, compared with risk-based screening at an HCV RNA positivity prevalence of 0.73%; sensitivity analyses generated an ICER of $50,000 per QALY gained or less until the prevalence of chronic hepatitis C infection dropped to 0.03%–0.04% (*92*).

Although real-world data informing screening during each pregnancy are lacking, a modeled analysis suggests that hepatitis C screening during each pregnancy would be cost-effective. Using a hepatitis C prevalence of 0.38% among pregnant women, as determined from national birth certificate data, the analysis found that universal hepatitis C screening during the first trimester of each pregnancy under a health care perspective compared with the current practice of risk-based screening had an ICER of $41,000/QALY gained (*93*). The model assumed no hepatitis C treatment would be offered until after 6 months postpartum and that 25% of women would be linked to care, with 92% of those linked initiating treatment. Only current injecting drug users were deemed at risk for new HCV infection or reinfection after cure. Universal screening reduced HCV-attributable mortality by 16% and more than doubled the proportion of infants born to mothers with hepatitis C who were identified as HCV-exposed, from 44% to 92%. ICER remained at or below $100,000 per QALY gained if hepatitis C prevalence was higher than 0.16%. This study did not account for any cost savings associated with prevention of risks for subsequent pregnancies or the potential benefits to early detection and management of infected infants.

### Hepatitis C Testing Strategy

The goal of hepatitis C screening is to identify persons who are currently infected with HCV. Hepatitis C testing should be initiated with a U.S. Food and Drug Administration (FDA)-approved anti-HCV test. Persons who test anti-HCV positive are either currently infected or had past infection that has resolved naturally or with treatment. Immunocompetent persons without hepatitis C risks who test anti-HCV negative are not infected and require no further testing. Persons testing anti-HCV positive should have follow-up testing with an FDA-approved nucleic acid test (NAT) for detection of HCV RNA. NAT for HCV RNA detection determines viremia and current HCV infection. Persons who test anti-HCV positive but HCV RNA negative do not have current HCV infection. CDC encourages use of reflex HCV RNA testing, in which specimens testing anti-HCV positive undergo HCV RNA testing immediately and automatically in the laboratory, using the same sample from which the anti-HCV test was conducted. Hepatitis C testing should be provided on-site when feasible.

### Determining the Prevalence Threshold for the Recommendations

The recommended HCV RNA prevalence threshold of 0.1% was determined based, in part, on review of published ICERs, as a function of hepatitis C prevalence, and the most up-to-date estimated prevalence of hepatitis C within states. In general, cost analyses determined that for all adults, ICER would be approximately $50,000 per QALY gained or less at current treatment costs (approximately $25,000 per course of treatment) at an anti-HCV positivity prevalence of 0.07% in the nonbirth cohort, which is similar to the HCV RNA prevalence in all adults. At a hepatitis C prevalence of 0.1%, ICER would be approximately $36,000 per QALY gained (*86*). Certain economists use $50,000 as a conservative threshold to determine cost-effectiveness. As treatment costs decrease, ICERs also will decrease, assuming other parameters remain stable. According to modeling results using NHANES data, no state has a hepatitis C prevalence in adults below 0.1% (*9*). Similarly, for universal testing in pregnant women, ICER would be approximately $50,000 per QALY gained or less at an HCV RNA positivity prevalence of 0.05%; at a prevalence of 0.1%, ICER would be approximately $15,000 per QALY gained (*92*). ICERs might be higher for testing in subsequent pregnancies when testing during the index pregnancy identifies women with hepatitis C who receive treatment following pregnancy, resulting in a decrease in hepatitis C prevalence among women with more than one pregnancy. According to birth certificate data (likely an underestimate of current maternal HCV infections), only three states were below the 0.1% prevalence among pregnant women (*4*).

Although the intent of public health screening is usually to identify undiagnosed disease, many persons previously diagnosed with hepatitis C are not appropriately linked to care and are not cured of their HCV infection, thereby representing an ongoing source of transmission. Therefore, the prevalence threshold of 0.1% should be determined on the basis of estimates of chronic hepatitis C prevalence, regardless of whether hepatitis C has been diagnosed previously.

## Recommendations

The following recommendations for hepatitis C screening augment those issued by CDC in 2012 (*5*). The recommendations issued by CDC in 1998 remain in effect (*6*). CDC recommends (Box 1):

BOX 1 **Persons recommended for hepatitis C testing**

**Table:** 

Universal hepatitis C screening: Hepatitis C screening at least once in a lifetime for all adults aged ≥18 years, except in settings where the prevalence of HCV infection (HCV RNA-positivity) is <0.1% Hepatitis C screening for all pregnant women during each pregnancy, except in settings where the prevalence of HCV infection (HCV RNA-positivity) is <0.1% One-time hepatitis C testing regardless of age or setting prevalence among persons with recognized risk factors or exposures: Persons with HIV Persons who ever injected drugs and shared needles, syringes, or other drug preparation equipment, including those who injected once or a few times many years ago Persons with selected medical conditions, including persons who ever received maintenance hemodialysis and persons with persistently abnormal ALT levels Prior recipients of transfusions or organ transplants, including persons who received clotting factor concentrates produced before 1987, persons who received a transfusion of blood or blood components before July 1992, persons who received an organ transplant before July 1992, and persons who were notified that they received blood from a donor who later tested positive for HCV infection Health care, emergency medical, and public safety personnel after needle sticks, sharps, or mucosal exposures to HCV-positive blood Children born to mothers with HCV infection Routine periodic testing for persons with ongoing risk factors, while risk factors persist: Persons who currently inject drugs and share needles, syringes, or other drug preparation equipment Persons with selected medical conditions, including persons who ever received maintenance hemodialysis Any person who requests hepatitis C testing should receive it, regardless of disclosure of risk, because many persons might be reluctant to disclose stigmatizing risks

- Universal hepatitis C screening (new recommendations):
  - Hepatitis C screening at least once in a lifetime for all adults aged ≥18 years, except in settings where the prevalence of HCV infection (HCV RNA-positivity) is <0.1%
  - Hepatitis C screening for all pregnant women during each pregnancy, except in settings where the prevalence of HCV infection (HCV RNA-positivity) is <0.1%
- One-time hepatitis C testing regardless of age or setting prevalence among persons with recognized conditions or exposures (existing recommendations):
  - Persons with HIV
  - Persons who ever injected drugs and shared needles, syringes, or other drug preparation equipment, including those who injected once or a few times many years ago
  - Persons with selected medical conditions, including persons who ever received maintenance hemodialysis and persons with persistently abnormal ALT levels
  - Prior recipients of transfusions or organ transplants, including persons who received clotting factor concentrates produced before 1987, persons who received a transfusion of blood or blood components before July 1992, persons who received an organ transplant before July 1992, and persons who were notified that they received blood from a donor who later tested positive for HCV infection
  - Health care, emergency medical, and public safety personnel after needle sticks, sharps, or mucosal exposures to HCV-positive blood
  - Children born to mothers with HCV infection
- Routine periodic testing for persons with ongoing risk factors, while risk factors persist:
  - Persons who inject drugs and share needles, syringes, or other drug preparation equipment
  - Persons with selected medical conditions, including persons who ever received maintenance hemodialysis
- Any person who requests hepatitis C testing should receive it, regardless of disclosure of risk, because many persons might be reluctant to disclose stigmatizing risks

### Determining Prevalence

In the absence of existing data for hepatitis C prevalence, health care providers should initiate universal hepatitis C screening until they establish that the prevalence of HCV RNA positivity in their population is <0.1%, at which point universal screening is no longer explicitly recommended but might occur at the provider’s discretion. Hepatitis C screening can be conducted in a variety of settings or programs that serve populations at different risk and with varying hepatitis C prevalence. Regardless of the provider, organization, or program providing testing, health care providers should initiate universal screening for adults and pregnant women unless the prevalence of HCV infection (HCV RNA positivity prevalence) in their patients has been documented to be <0.1%. There are statistical challenges with determining a “number needed to screen” to detect a relatively rare disease in lower-risk settings; therefore, providers and program directors are encouraged to consult their state or local health departments or CDC to determine a reasonable estimate of baseline prevalence in their setting or a methodology for determining how many persons they need to screen before confidently establishing that the prevalence is <0.1%. As a general guide: as HCV RNA prevalence is predicated on first testing for anti-HCV, and according to the most current serologic data in the United States, approximately 59% of anti-HCV positive persons are HCV RNA positive (*2*), an estimated 507 randomly selected patients in a setting of any size would need to be tested using any of the available anti-HCV tests (*94*) to detect an anti-HCV prevalence positivity of ≤0.17%, corresponding to an expected HCV RNA positivity prevalence of 0.1% with 95% confidence and 5% tolerance (*95*) (https://epitools.ausvet.com.au).

### Patient Follow-up After Hepatitis C Testing

Providers and patients can discuss hepatitis C screening as part of a person’s preventive health care. For persons identified with current HCV infection, CDC recommends that they receive appropriate care, including hepatitis C-directed clinical preventive services (e.g., screening and intervention for alcohol or drug use, hepatitis A and hepatitis B vaccination, and medical monitoring of disease).

Recommendations are available to guide management of persons infected with HCV (Box 2). Persons infected with HCV can benefit from treatment, prevention, and other counseling messages.

BOX 2 **Management of persons with HCV infection**

**Table:** 

Medical evaluation (by either a primary-care clinician or specialist [e.g., in hepatology, gastroenterology, or infectious disease]) for chronic liver disease, including treatment and monitoring Hepatitis A and hepatitis B vaccination Screening and brief intervention for alcohol consumption Avoiding new medicines, including over-the-counter and herbal agents, without first checking with their health care provider HIV risk assessment and testing Weight management or losing weight and following a healthy diet and staying physically active for persons who are overweight (BMI ≥25kg/m^2^) or obese (BMI ≥30kg/m^2^) Avoiding or stopping donating blood, tissue, or semen Refraining from sharing appliances that might come into contact with blood, such as toothbrushes, dental appliances, razors, nail clippers, glucose meters, and lancet devices.

- Persons with negative anti-HCV test results should be informed of their test results and reassured that they are not infected, unless they were recently exposed to infection (e.g., recent IDU). Repeat testing should occur for persons with ongoing risk behaviors. Persons with negative anti-HCV and positive HCV RNA test results have recent HCV infection.
- Persons with positive anti-HCV and negative HCV RNA test results should be informed that they do not have current HCV infection. Test results indicate either a resolved past infection or a false-positive anti-HCV test result. Additional testing might be warranted to determine the patient’s status.
- Persons with positive anti-HCV and positive HCV RNA test results should be informed that they have active HCV infection and would benefit from curative treatment. They will need further evaluation before treatment, medical care for possible liver disease, and ongoing medical monitoring. Persons with HCV infection should be provided information about treatment options, how to prevent transmission of HCV to others, and drug treatment, as appropriate. Persons with hepatitis C also should be informed about the resources available to them within their communities, including providers of medical evaluation, harm reduction, and social support.
  - At the time when positive test results are communicated to patients, health care providers should evaluate the patient’s level of alcohol and drug use and provide a brief alcohol or drug use intervention, if clinically indicated (*5*).

### Testing Considerations

Universal hepatitis C screening was compared with risk-based screening for adults and pregnant women. As such, the marginal benefits and harms of universal screening compared with birth cohort screening was not directly assessed. For the purposes of this literature review, the birth cohort was deemed a risk group, and studies comparing birth cohort with universal screening strategies were eligible for inclusion. The incidence of acute hepatitis C is greatest among persons younger than birth cohort members (*5*). Because most pregnant women are younger than persons born during the 1945–1965 birth cohort, hepatitis C testing among pregnant women has previously been based on the presence of risk factors. The new recommendations apply to all pregnant women, including those aged <18 years.

Data informing the optimal time during pregnancy for hepatitis C testing are lacking. If DAA treatment becomes available for use during pregnancy, testing at an early prenatal visit would allow for identification of women who could benefit from treatment. Testing early in pregnancy also could inform pregnancy and delivery management per the Society for Maternal-Fetal Medicine recommendations for a preference for amniocentesis over chorionic villus sampling and for avoiding internal fetal monitoring, prolonged rupture of the membranes, and episiotomy (*44*). Testing at an early prenatal visit harmonizes testing for hepatitis C with testing for other infectious diseases during pregnancy; however, this strategy might miss women who acquire HCV infection later during pregnancy. Pregnant women with ongoing risk factors tested early in pregnancy could undergo repeat testing later in pregnancy to identify those who acquired HCV infection later in pregnancy. Hepatitis C screening during pregnancy should be an opportunity to promote a dialogue between the pregnant woman and her medical provider about hepatitis C transmission and risk factors.

Hepatitis C prevalence in U.S. correctional settings is high because of high incarceration rates among persons who use drugs (*96*). Two recent systematic reviews estimated average anti-HCV positivity prevalence in correctional settings at 16.1% and 23% (*2*,*97*). Hepatitis C prevalence varies across individual correctional jurisdictions based on factors including underlying community prevalence, sentencing standards for drug-related offenses, and type of institution. These estimates exceed both the general population prevalence of 1.7% (*2*) and the target threshold of ≥0.1% at which these guidelines recommend universal hepatitis C testing in other settings. Therefore, the well-documented prevalence of HCV infection in a variety of correctional jurisdictions supports the application of these guidelines to prisons and jails. Universal hepatitis C testing in correctional facilities can be expected to yield higher infection identification rates compared with the risk-based testing practices that many jurisdictions employ (*98*,*99*) and to support broader hepatitis C elimination efforts (*34*,*100*,*101*).

### Reporting

Cases of hepatitis C should be reported to the appropriate state or local health jurisdiction in accordance with requirements for reporting acute, perinatal, and chronic HCV infection. Case definitions for the classification of reportable cases of HCV infection have been published previously by the Council of State and Territorial Epidemiologists (*102*).

### Recommendations of Other Organizations

Recommendations in this report for hepatitis C screening among certain groups differ somewhat from the recommendations of other organizations. The U.S. Preventive Services Task Force (*103*) and AASLD and IDSA (*39*) also make recommendations for hepatitis C testing.

## Future Directions

CDC will review and possibly revise these recommendations as new epidemiology or other information related to hepatitis C becomes available, including potential availability of DAA treatments for pregnant women, infants, and younger children, and the experience gained from the implementation of these recommendations. A review of the evidence regarding infant testing is needed to inform future recommendations for an infant testing algorithm. Evidence should examine the benefits and harms of HCV RNA testing beginning at age 2 months compared with anti-HCV testing at or after age 18 months. The greater expense of HCV RNA testing might be justified as earlier testing will likely minimize loss to follow-up. Additional data on the safety of DAA use during pregnancy are needed to inform treatment during pregnancy, which might reduce the risk for perinatal transmission. Finally, for expanded screening to be effective in reducing the morbidity and mortality of hepatitis C in the United States, models to address barriers related to access to DAA treatment are needed.

## Conclusion

CDC recommends hepatitis C screening of all adults aged ≥18 years once in their lifetimes, and screening of all pregnant women (regardless of age) during each pregnancy. The recommendations include an exception for settings where the prevalence of HCV infection is demonstrated to be <0.1%; however, few settings are known to exist with a hepatitis C prevalence below this threshold (*2*,*9*). The recommendation for testing of persons with risk factors remains unchanged; those with ongoing risk factors should be tested regardless of age or setting prevalence, including continued periodic testing as long as risks persist. These recommendations can be used by health care professionals, public health officials, and organizations involved in the development, implementation, delivery, and evaluation of clinical and preventive services.
